# DGKα Enhances Tumorigenic Activity in Bladder Cancer Patients With Chronic Kidney Disease

**DOI:** 10.1002/cam4.70710

**Published:** 2025-02-27

**Authors:** Kenshiro Takemoto, Kohei Kobatake, Tomoya Hatayama, Shinsaku Tasaka, Mai Okazaki, Yoshinori Nakano, Hiroyuki Shikuma, Kazuma Yukihiro, Kyosuke Iwane, Ryoken Yamanaka, Ryo Tasaka, Yuki Kohada, Miki Naito, Shunsuke Miyamoto, Yohei Sekino, Hiroyuki Kitano, Keisuke Goto, Akihiro Goriki, Keisuke Hieda, Nobuyuki Hinata

**Affiliations:** ^1^ Department of Urology Graduate School of Biomedical and Health Sciences, Hiroshima University Hiroshima Japan

**Keywords:** bladder cancer, chronic kidney disease, tumorigenic activity

## Abstract

**Introduction:**

Chronic kidney disease (CKD) is a risk factor for bladder cancer (BC) and is reportedly involved in its recurrence and progression. This study aimed to determine the molecular mechanisms underlying the development of BC in patients with CKD.

**Methods:**

First, we generated the CKD mouse model according to a unilateral two‐stage renal ischemia–reperfusion injury protocol using wild‐type C57BL/6 mice. Second, we conducted a molecular functional analysis of DGKα in BC and investigated the contribution of DGKα to cell invasion, migration, and proliferation activity using human BC cell lines.

**Results:**

After confirming elevated serum creatinine levels in mice, the bladder was dissected, and mRNA sequencing of bladder urothelial cells was conducted. Gene expression profiling revealed remarkable upregulation in diacylglycerol kinase alpha (DGKα) level compared to that in control urothelial cells. DGKα‐knockdown cells displayed significantly decreased invasion, migration, and proliferation activity compared to the controls. Next, we conducted a clinical analysis of DGKα in BC patients and performed immunohistochemistry (IHC) on samples from patients treated with radical cystectomy. IHC staining revealed that DGKα‐positive cases had significantly worse recurrence‐free and cancer‐specific survival rates (*p* = 0.036 and = 0.003, respectively).

**Conclusion:**

DGKα expression is associated with tumorigenic activity in BC. Therefore, it is speculated that increased expression of DGKα in CKD cases is involved in the malignant potentials in BC. In conclusion, the crucial role of DGKα in BC is suggested, and it may be one of the factors contributing to poor prognosis in BC patients with CKD.

**Abbreviations**
BC: bladder cancer
BUN: blood urea nitrogen
CKD: chronic kidney disease
DGKα: diacylglycerol kinase alpha
IRI: ischemia–reperfusion injury
PA: phosphatidic acid

## 1 Introduction

Bladder cancer (BC), a common urological malignancy of the bladder urothelium, is the ninth‐most commonly diagnosed cancer worldwide, with approximately 613,000 new cases and 220,000 deaths reported in 2022 [1]. Advanced age is the greatest risk factor for BC, and this is explained by exposure to carcinogens such as cigarette smoke and, less commonly, benzene chemicals and aromatic amines, combined with an age‐related reduction in the ability to repair DNA [2]. Other risk factors include bacterial and parasitic infections, chronic indwelling Foley catheter use, prior bladder augmentation, and pelvic radiation. These risk factors are mostly associated with chronic inflammatory processes and are strongly linked to each other, and consequently, they play a crucial role in cancer progression.

Chronic kidney disease (CKD) is a representative disease related to chronic inflammation in the systemic and urinary systems. Our previous study reported that CKD is an independent predictor of recurrence and progression in primary non‐muscle‐invasive BC [3]. Recently, various reports have revealed the important role of CKD in BC recurrence and progression, not only in non‐muscle‐invasive BC but also in muscle‐invasive BC [4, 5]. Although it is becoming clear that CKD, a non‐specific phenomenon related to various factors in vivo, is a risk and poor prognostic factor for BC, the molecular mechanisms underlying the effects of CKD on the tumorigenicity of BC have not been clarified.

In this study, we searched for tumorigenic genes associated with CKD. Remarkable upregulation of diacylglycerol kinase alpha (DGKα) was evaluated in the CKD mouse model bladder urothelium. DGKα acts as a modulator that competes with protein kinase C for the second messenger diacylglycerol in intracellular signaling pathways. It also plays an important role in the synthesis of phosphatidic acid (PA), an intracellular signaling molecule, by phosphorylating diacylglycerols. DGKα has been shown to have relations with the activation of HIF‐1α, c‐MET, ALK, and VEGF [6, 7, 8, 9]. Herein, we report a novel role of DGKα in BC and that the expression of DGKα is closely related to the poor prognosis of patients with BC treated with radical cystectomy, in association with its enhanced invasion, migration, and proliferation activity in BC cell lines. Furthermore, via DGK‐mediated pathways, there is a possibility that DGKα becomes a therapeutic biomarker and new therapeutic target in BC.

## 2 Material and Methods

### 2.1 CKD Model Mice

To generate CKD mouse model, we induced a “unilateral two‐stage ischemia–reperfusion injury (IRI)” in 8‐week‐old male C57BL/6J mice (Charles River) using a previously reported method [10]. At the beginning of Stage I (Day 1), IRI was performed by clamping the left renal pedicle with a silver clip for 24 min. At the time, the right kidney remained intact. The wound was sutured after the release of the clip, and the mice were allowed to recover for 14 d. The right kidney was removed at the beginning of Stage II (Day 14). Mice that underwent right kidney nephrectomy on Day 14, but not left renal pedicle clamping on Day 1, served as controls. For the next 70 days, the mice were maintained in an appropriate environment. Eighty‐four days after the start of this experiment, after confirming elevated serum blood urea nitrogen (BUN) and creatinine levels, the mice were sacrificed, and their bladder urothelium was excised under a microscope. Total RNA excised from the bladder urothelium was converted into libraries using a SureSelect Strand‐Specific RNA Library Preparation Kit (Agilent Technologies, Santa Clara, CA, USA). Transcriptome analysis was performed using the next‐generation sequencer HiSeq 2500 (Illumina, San Diego, CA, USA). In this RNA sequencing analysis, the CKD and control groups were compared using two mice each. All animal experiments were performed in strict accordance with the recommendations of the Guide for the Care and Use of Laboratory Animals of Hiroshima University Animal Research Committee (permission nos. 29–58).

### 2.2 RNA Sequencing Analysis and GSEA

Total RNA extracted from the excised bladder urothelium of the CKD model and control mice was converted into libraries using the SureSelect Strand‐Specific RNA Library Preparation kit (Agilent Technologies). The next‐generation sequencer HiSeq 2500 (Illumina) was used for transcriptome analysis. The generated sequence tags were mapped to human genomic sequences (hg38). Gene Set Enrichment Analysis software (version 4.1.0) was downloaded from the websites of the Broad Institute (San Diego, CA, USA; http://software.broadinstitute.org/gsea/downloads.jsp).

### 2.3 Tissue Samples

Forty‐two patients diagnosed with BC who underwent radical cystectomy without neoadjuvant chemotherapy at Hiroshima University Hospital between April 1999 and May 2011 were included in this study. Patients who had received neoadjuvant chemotherapy were excluded because of the associated renal dysfunction. Pathological diagnoses were made based on the World Health Organization (WHO) classification [11]. Tumor grade was classified according to the 1973 WHO grading system. Upon evaluating the patients' backgrounds and survival prognoses, we obtained relevant clinicopathological data from medical records, such as age, sex, pathological tumor, node, metastasis (TNM) stage, tumor grade, and blood biochemical tests. Tumor staging was performed according to the 2010 American Joint Committee on Cancer TNM Staging System [12]. This study was conducted in accordance with the guidelines of the Declaration of Helsinki and Good Clinical Practice. All experimental procedures were approved by the Ethics Committee of the Hiroshima University Hospital (approval no. E‐326‐2). All patients provided written informed consent before participation.

### 2.4 Immunohistochemical Staining

Immunohistochemical (IHC) staining was performed on tumor sections obtained by radical cystectomy as described previously [13]. IHC staining analysis was performed with a Dako Envision+ Mouse Peroxidase Detection System (Dako Cytomation, Carpinteria, CA, USA). Antigen retrieval was performed by microwave heating in citrate buffer (pH 8.0) for 1 h. Peroxidase activity was blocked with 3% H_2_O_2_‐methanol for 5 min, and the sections were incubated with normal goat serum (Dako Cytomation) for 10 min to block nonspecific antibody binding sites. Sections were incubated with a rabbit polyclonal anti‐DGKα antibody (11547‐1‐AP; Proteintech Group, Rosemont, IL, USA, 1:200) for 1 h at room temperature, followed by incubation with Envision+ anti‐rabbit or ‐mouse peroxidase for 1 h. The sections were incubated with DAB Substrate‐Chromogen Solution (Dako Cytomation) for 5 min for color reaction and then counterstained with 0.1% hematoxylin. DGKα expression in BC was scored in all tumors as positive or negative, upon blind examination by two independent uropathologists.

### 2.5 Cell Culture

Human BC cell lines T24 (Research Resource Identifier (RRID) number: CVCL_0554), 5637 (CVCL_0126), RT‐4 (CVCL_0036), RT‐112 (CVCL_1670), UM‐UC‐3 (CVCL_1783), and UM‐UC‐13 (CVCL_2746) were purchased from the Japanese Collection of Research Bioresources Cell Bank (Osaka, Japan) and maintained at 37°C in a humidified atmosphere containing 5% CO_2_ in RPMI‐1640 medium (Sigma‐Aldrich, St. Louis, MO, USA) supplemented with 10% fetal bovine serum (FBS) (Gibco, Paisley, UK) and 1% penicillin–streptomycin (FUJIFILM Wako Pure Chemical Corporation, Osaka, Japan).

### 2.6 Transfection and RNA Interference

To knock down DGKα expression in T24, 5637, RT‐4, RT‐112, UM‐UC‐3, and UM‐UC‐13 cells, we used small interfering RNA (siRNA)‐based RNA interference technology. Cells were independently transfected with two different siRNAs against human DGKα (si DGKα‐1; #s3912 and si DGKα‐2, #s3913; Stealth RNAi; Thermo Fisher Scientific, Waltham, MA, USA) or control siRNA (siCtrl; Silencer; Thermo Fisher Scientific) using Lipofectamine RNAiMAX reagent (Thermo Fisher Scientific). Cells were collected 48 and 72 h after RNA and protein extraction, respectively.

### 2.7 Quantitative RT‐PCR Analysis

Quantitative RT‐PCR was performed as previously described [13]. Total RNA was isolated using NucleoSpin RNA (Takara, Japan), and 500 ng of total RNA was reverse‐transcribed into cDNA using PrimeScript RT Master Mix (Takara). Quantitative PCR (qPCR) was performed for cDNA with SYBR Select Master Mix (Applied Biosystems, Austin, TX, USA) using the StepOnePlus Real‐Time PCR System (Applied Biosystems). The cycle threshold (Ct) values were normalized to the expression of an endogenous housekeeping gene, hypoxanthine phosphoribosyltransferase (HPRT), and 2^(−ΔΔCt)^ values were calculated for relative quantification. Reactions were performed in triplicate using the qPCR primers listed in Table S1.

### 2.8 Western Blotting Analysis

Western blotting was performed as previously described [13]. Briefly, protein samples were electrophoresed on 5%–20% precast polyacrylamide gels (SuperSep Ace; FUJIFILM Wako Pure Chemical Corporation) and transferred onto nitrocellulose blotting membranes (GE Healthcare Life Science, Uppsala, Sweden) by electroblotting. Membranes were blocked with 5% nonfat dry milk in TBST (10 mM Tris, 150 mM NaCl, and 0.05% Tween 20; pH 8.0) for 30 min and incubated with primary antibodies at 4°C overnight. The primary antibodies used in these assays were as follows: anti‐human DGKα (1:1000; 11,547‐1‐AP; Proteintech Group) and anti‐β‐actin (1:5000; #A2228; Sigma‐Aldrich).

### 2.9 Transwell Invasion Assay

We evaluated the cell invasion ability using a transwell invasion assay. For this assay, 24‐well cell culture inserts (Corning, NY, USA) and 8.0‐μm pore size ThinCerts membranes (Greiner Bio‐One, Kremsmunster, Austria) were used. Cells suspended in serum‐free medium were seeded into the top chamber after serum‐containing medium (10% FBS) was added to the bottom chamber. Twenty‐four hours later, the cells that translocated to the bottom surface of the membrane were recovered and counted using Diff‐Quick staining.

### 2.10 Wound‐Healing Assay

We conducted a wound‐healing assay to evaluate cell migration. Cells were seeded into a culture insert (ibidi Culture‐Insert 2 Well; ibidi GmbH, Martinsried, Germany) at a density of 5.0 × 10^5^ cells/mL. After allowing the cells to adhere overnight, the culture insert was recovered and washed with PBS to remove the non‐adherent cells. The cells were then cultured in fresh medium for 10 h. After photographing the plate at the beginning and end of the culture period, the cells that had migrated into the wound space were manually enumerated in three fields per well under a light microscope at ×50 magnification. Cell migration areas were quantified using the ImageJ software (NIH, Bethesda, MD, USA; https://imagej.nih.gov/ij/).

### 2.11 Cell Proliferation Assay

Cells (5.0 × 10^3^/mL) were incubated in a 96‐well cell culture plate for 24 h. Cell proliferation was assessed using the Premix WST‐1 Cell Proliferation Assay System (Takara) and expressed as the absorbance measured at 450 nm using a microplate reader. For each measurement, wells with untreated cells and media without cells served as controls and blanks, respectively.

### 2.12 Statistical Analyses

Multiple group comparisons were performed using one‐way ANOVA with Dunnett's multiple comparison test. Statistical analyses were performed using GraphPad Prism 8 software (GraphPad Software Inc., San Diego, CA, USA; RRID:SCR_002798). Linear regression analysis, Fisher's exact test, unpaired *t*‐test, *χ* ^2^ test, and log‐rank test were performed to compare the two groups. Univariate and multivariate Cox regression analyses were performed using JMP software (SAS Institute Inc., Cary, USA; RRID:SCR_008567). *p*‐values less than 0.05 were considered to indicate statistically significant differences.

## 3 Results

### 3.1 Generation of CKD Model Mouse and RNA Sequencing Analysis

**FIGURE 1:**
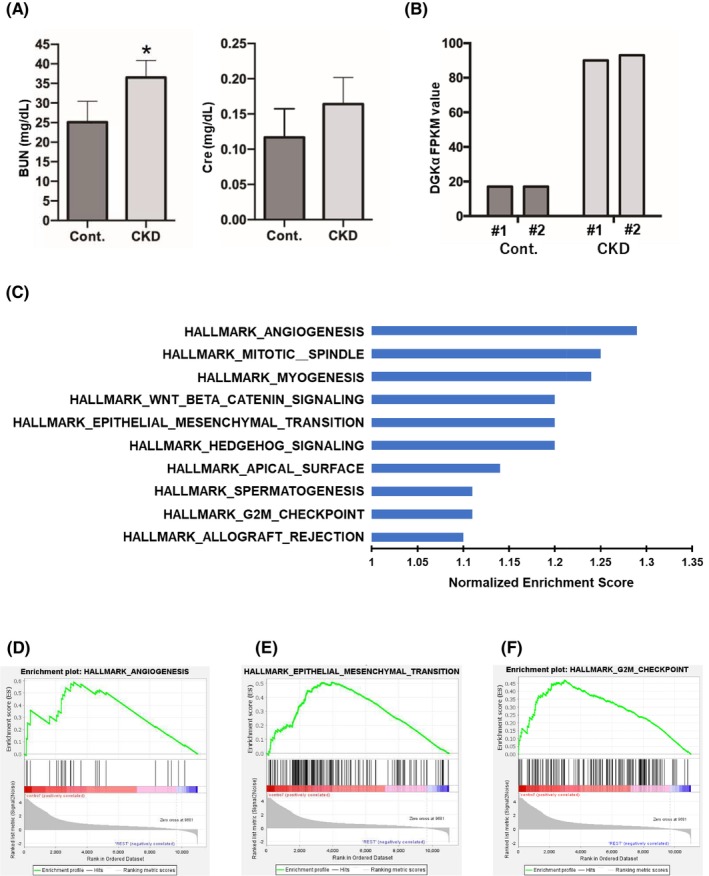
Generation of chronic kidney disease (CKD) model mice and Gene Set Enrichment Analysis (GSEA) of the bladder urothelium of CKD mice. (A) The elevation of serum blood urea nitrogen and creatinine in CKD model mice. (B) The upregulation of DGKα expression in the bladder urothelium of CKD mice compared with control mice. (C) Normalized enrichment scores of the top 10 upregulated pathways of hallmark gene sets enriched in the bladder urothelium of CKD mice compared with control mice. (D–F) Enrichment pattern of hallmark angiogenesis rerated (D), hallmark epithelial‐mesenchymal transition related (E), and G2M checkpoint‐related (F) gene sets determined by GSEA comparing CKD and control mice.

First, the CKD mouse model was generated according to the “unilateral two‐stage IRI” protocol. For the unilateral IRI (uIRI) time, we used the 24‐min protocol that resulted in continuous increases in creatinine levels and declines in the glomerular filtration rate. The survival rate associated with this procedure was approximately 80%. The dead mice exhibited marked edema throughout their bodies, suggesting that the cause of death was likely renal failure. At the end of the experiments (Day 84), we collected blood samples from the surviving mice, performed blood tests, and measured serum BUN and creatinine levels. After confirming elevated serum BUN and creatinine levels (Figure 1A), the mice were euthanized and only the bladder urothelium was excised under a microscope. Total RNA was extracted from the excised bladder urothelium, and gene expression profiles were evaluated via mRNA sequencing analysis. Among the different upregulated genes, we especially examined DGKα, which had the least gene expression level dispersion within each group, and previous studies have suggested its involvement in tumorigenic activity in other types of carcinomas. DGKα was remarkably upregulated in the bladder urothelium of CKD mice compared to that of the control (Figure 1B). RNA sequencing was performed on the excised bladder urothelium. A series of hallmark gene sets were extracted from the GSEA (Figure 1C). Several notable pathways were identified, including angiogenesis, epithelial–mesenchymal transition (EMT), and G2M checkpoint pathways (Figure 1D–F).

### 3.2 Expression of DGKα in Patients With BC Treated With Radical Cystectomy

**FIGURE 2:**
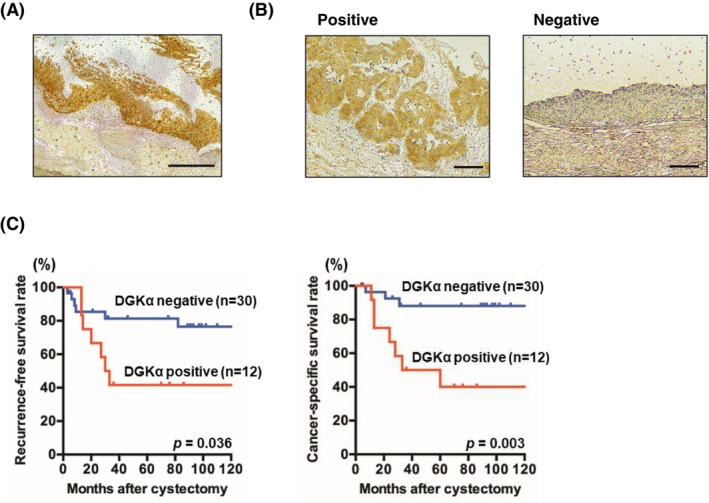
Relationship between DGKα expression and bladder cancer (BC) patients treated with radical cystectomy. (A) Immunohistochemical DGKα staining in BC patients. The results shown are representative of 42 independent specimens. Original magnification, ×100. Scale bar, 200 μm. (B) Immunohistochemical staining of DGKα‐positive and ‐negative tumors. The results shown are representative of 12 DGKα‐positive and 30 DGKα‐negative specimens. Original magnification, ×400. Scale bar, 50 μm. (C) Kaplan–Meier plots of recurrence‐free (left) and cancer‐specific (right) survival rates for DGKα‐positive and ‐negative BC patients. *p*‐values calculated by a log‐rank test are also indicated.

Expression levels of DGKα in tumor specimens from patients with BC treated with radical cystectomy were analyzed. All patients are pure urothelial carcinoma, with no cases containing histological variants. IHC staining patterns of BC specimens were classified as positive or negative based on the judgment of independent uropathologists. Weak or no staining of DGKα was observed in non‐neoplastic urothelium, whereas stronger and more extensive staining was observed in BC tissues (Figure 2A). Even in tumor samples, DGKα‐positive and ‐negative specimens were evidently distinguishable (Figure 2B). Staining of DGKα was mainly observed in the cytoplasm in BC. Twelve (29%) of the 42 tumor specimens were positive for DGKα. Kaplan–Meier analysis revealed worse recurrence‐free survival and cancer‐specific survival rates in DGKα‐positive patients compared to DGKα‐negative patients (*p* = 0.036 and = 0.003, respectively) (Figure 2C). On the other hand, DGKα expression was not associated with pathologic T stage and grade; however, lymph node metastasis rate was significantly higher in DGKα‐positive patients. Furthermore, the estimated glomerular filtration rate (eGFR) tended to be lower in the DGKα‐positive group (Table 1).

**TABLE 1:** Comparison of clinical characteristics between DGKα‐positive and ‐negative BC patients treated with radical cystectomy.

	Case (% of total)		*p*
	DGKα‐positive (*n* = 12)	DGKα‐negative (*n* = 30)	
Age, years			
≤ 70	7 (58.3)	15 (50.0)	0.62
> 70	5 (41.7)	15 (50.0)	
Gender			
Male	9 (75.0)	24 (80.0)	0.72
Female	3 (25.0)	6 (20.0)	
eGFR, mL/min/1.73 m^2^			
≥ 45	8 (66.7)	25 (83.3)	0.24
< 45	4 (33.3)	5 (16.7)	
Pathologic T stage			
< pT3	7 (58.3)	22 (73.3)	0.35
≥ pT3	5 (41.7)	8 (26.7)	
Tumor Grade			
G1/2	3 (25.0)	5 (16.7)	0.54
G3	9 (75.0)	25 (83.3)	
Lymph node status			
N0	9 (75.0)	29 (96.7)	0.04
N1	3 (25.0)	1 (3.3)	

### 3.3 DGKα Promotes Cell Invasion, Migration, and Proliferation Activities In Vitro

**FIGURE 3:**
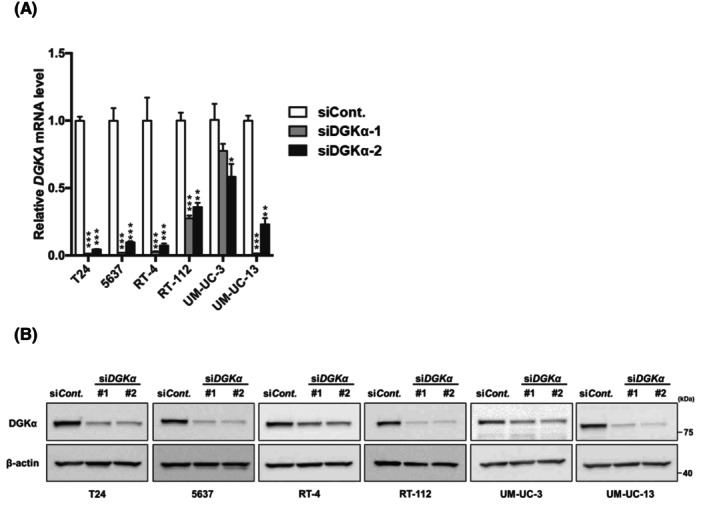
Successful knocking down of DGKα in BC cell lines. (A) DGKα‐knockdown (siDGKα‐1 and siDGKα‐2) BC cell lines were subjected to determine mRNA levels of DGKα by quantitative RT‐PCR analysis. Results are expressed as the mean ± SEM or representative of three experiments. *p < 0.05, **p < 0.01, and ***p < 0.001 by one‐way ANOVA combined with Dunnett's multiple comparisons test, compared with siCont. siCont, control siRNA‐introduced control. (B) DGKα‐knockdown (siDGKα‐1 and siDGKα‐2) BC cell lines were subjected to determine protein levels of DGKα by western blotting analysis.

**FIGURE 4:**
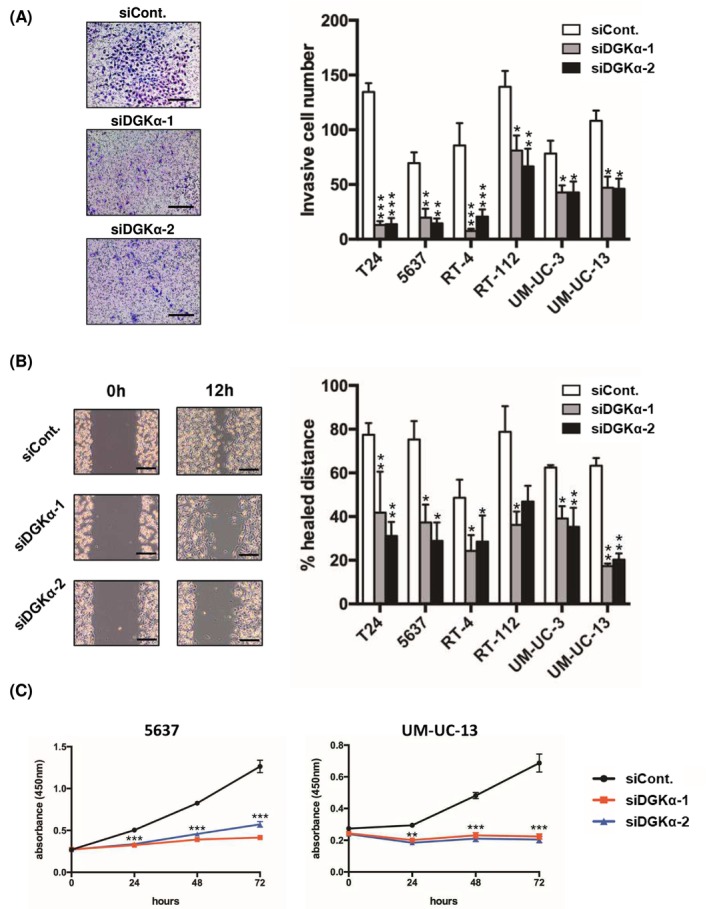
Role of DGKα in BC cell invasion, migration, and proliferation in vitro. (A) DGKα‐knockdown cell lines were subjected to transwell invasion assay with corresponding control cells (siCont) (*n* = 4). *p < 0.05, **p < 0.01, and ***p < 0.001 by one‐way ANOVA combined with Dunnett's multiple comparisons test, compared with siCont. Representative Diff‐Quick‐stained transwell membranes using T24 cells were shown in left panels. Scale bar, 50 μm. (B) DGKα‐knockdown cell lines were subjected to a wound healing assay with corresponding control cells (siCtrl) (*n* = 3). *p < 0.05 and **p < 0.01 by one‐way ANOVA combined with Dunnett's multiple comparisons test, compared with siCont. Representative images of migrated cells using T24 cells were shown in the left panels. Scale bar, 50 μm. (C) DGKα‐knockdown cell lines were subjected to a cell proliferation assay with corresponding control cells (siCont) (*n* = 4). **p < 0.01 and ***p < 0.001 by one‐way ANOVA combined with Dunnett's multiple comparisons test, compared with siCont.

To determine the functional significance of DGKα in BC, we investigated the contribution of DGKα to cell invasion, migration, and proliferation by knocking down DGKα in the following well‐known BC cell lines: T24, 5637, RT‐4, RT‐112, UM‐UC‐3, and UM‐UC‐13 cells. Successful knockdown of DGKα was confirmed at the mRNA (Figure 3A) and protein (Figure 3B) levels using qRT‐PCR and western blotting, respectively. We investigated the effect of DGKα knockdown on cell invasion, migration, and proliferation activities to determine the functional significance of DGKα in BC. First, we performed a Transwell invasion assay to measure cell invasion activity, which revealed that DGKα‐knockdown cells displayed significantly decreased invasive activity compared to control cells (Figure 4A). Second, a wound‐healing assay was performed to measure cell invasion activity. Consistent with the Transwell invasion assay results, the migration activity of BC cells was downregulated by knockdown of DGKα (Figure 4B). Third, we evaluated cell proliferation activity using the WST‐1 assay, which revealed that knockdown of DGKα decreased cell proliferation activity in 5637 and UM‐UM‐13 cells (Figure 4C).

## 4 Discussion

CKD is a poor prognostic factor in patients with BC [14]. However, the molecular mechanisms underlying the effects of CKD in BC have not been thoroughly investigated. This is the first study to clarify the critical contribution of CKD to BC using a CKD mouse model. In the CKD mouse model, the survival rate of the uIRI‐24 min group was 75%, consistent with a previous study [10]. Consistent with the results of this study, during the process of generating CKD mouse models, the survival rate was almost 80%. Although there are likely to be a certain number of deaths due to nephrectomy invasion, the dead mice showed marked edema throughout the body, suggesting that the cause of death was renal failure. Only surviving mice could be used for the analysis, which may explain why there were no significant differences in elevated serum creatinine levels. RNA sequencing analysis revealed a remarkable upregulation of DGKα, an enzyme that converts the membrane lipid diacylglycerol to phosphatidic acid, in the bladder urothelium of CKD mice. Hence, we focused on DGKα and evaluated its functional role in BC. The expression of DGKα was correlated with poor prognosis in patients with BC treated with radical cystectomy. Furthermore, DGKα significantly contributes to the increased invasion, migration, and proliferation ability of BC cell lines in vitro.

The relationship between DGKα expression and cancer progression has been reported in several cancers, including hepatocellular carcinoma, melanoma, glioblastoma, colon adenocarcinoma, and breast adenocarcinoma [15, 16, 17, 18]. In this report, we showed the potential utility of CKD‐induced DGKα upregulation and its corresponding molecular mechanisms in predicting the prognosis of patients with BC. Expression of DGKα was found to be deeply involved in tumorigenic activity through its involvement in invasion, migration, and proliferation activity. These results provide a reasonable explanation for our clinical findings indicating that CKD is a risk factor for recurrence and progression in patients with primary non‐muscle‐invasive BC [19] and for other reports suggesting that patients with CKD have a poor prognosis after radical cystectomy [20].

Li et al. [21] reported that DGKα elicits platinum resistance in ovarian cancer. In particular, they clarified that loss of DGKα selectively ameliorates cisplatin sensitivity in cisplatin‐resistant ovarian cancer in a kinase‐dependent manner via its metabolic product, PA. PA activates the transcription factor c‐JUN by binding to it and translocating it into the nucleus to promote WEE1 expression. WEE1 is a regulator of cell cycle progression upon cisplatin exposure, which regulates the G2 checkpoint and prevents entrance into mitosis in response to DNA damage [22, 23]. This DGKα–c‐JUN–WEE1 signaling pathway reportedly provides cisplatin resistance in ovarian cancer. Considering its involvement in the G2M checkpoint pathways observed in the RNA sequencing analysis in this study, DGKα may have been considered to contribute to cisplatin resistance in BC through WEE1. Platinum‐based compounds, such as cisplatin or carboplatin, are standard chemotherapy agents in patients with BC and have been commonly used for decades. There is a possibility that the activation of DGKα induces platinum resistance in patients with CKD and significant poor prognosis in BC patients with CKD. Furthermore, Fu et al. [24] clarified that DGKα phosphorylated the proto‐oncogene tyrosine‐protein kinase Src (SRC) protein and focal adhesion kinase 1 (FAK) protein to form and activate the DGKα/SRC/FAK complex, thereby initiating the downstream WNT/β‐catenin and VEGF signaling pathways, promoting EMT and angiogenesis, and resulting in the metastasis of non‐small cell lung cancer. Consistent with this result, GSEA in this study revealed an association between EMT and angiogenesis pathways. This molecular mechanism might reasonably explain the significantly higher lymph node metastasis rate in DGKα‐positive patients treated with radical cystectomy in our clinical cohort. Furthermore, they additionally reported that DGKα also mediates PD‐1 blockade [25]. Currently, in BC, drug therapy using immune checkpoint inhibitors, represented by PD‐1 antibodies, is the mainstream treatment. This suggests that DGKα may become not only a prognostic predictor for BC through CKD but also a new potential therapeutic target. BC is a diverse disease concept ranging from low to high grade. Although molecular mechanisms and prognostic indicators have been reported [26, 27], the pathogenesis of BC remains largely unknown.

Our study had certain limitations. First, in the IHC analysis, DGKα‐positive cases tended to exhibit reduced kidney function compared to DGKα‐negative cases, although no significant difference was observed. This may be owing to the fact that only a small number of cases treated with radical cystectomy were included in the retrospective analysis. Therefore, a prospective study using a large number of patients with BC will be necessary to verify the present data. Second, the mechanisms underlying the upregulation of DGKα in the bladder urothelium of CKD mice remain unclear.

In summary, a significant upregulation of DGKα was observed in CKD mouse models. The expression of DGKα was associated with poor prognosis of patients with BC treated with radical cystectomy. In addition, the DGKα‐mediated cellular invasion, migration, and proliferation activity was suggested. DGKα could be developed as a predictor for BC patients with CKD and potential new therapeutic targets.

## Author Contributions

**Kenshiro Takemoto:** conceptualization, methodology, data curation, investigation, writing – original draft, project administration. **Kohei Kobatake:** conceptualization, methodology, data curation, supervision, investigation, project administration. **Tomoya Hatayama:** data curation, investigation. **Shinsaku Tasaka:** data curation. **Mai Okazaki:** data curation. **Yoshinori Nakano:** data curation. **Hiroyuki Shikuma:** data curation. **Kazuma Yukihiro:** data curation. **Kyosuke Iwane:** data curation. **Ryoken Yamanaka:** data curation, investigation. **Ryo Tasaka:** data curation, investigation. **Yuki Kohada:** data curation, investigation. **Miki Naito:** data curation. **Shunsuke Miyamoto:** data curation. **Yohei Sekino:** data curation, investigation, project administration. **Hiroyuki Kitano:** data curation, investigation. **Keisuke Goto:** data curation, investigation, project administration. **Akihiro Goriki:** data curation, investigation. **Keisuke Hieda:** data curation, investigation. **Nobuyuki Hinata:** project administration, supervision, writing – review and editing.

## Ethics Statement

Approval of the research protocol by an Institutional Reviewer Board. All experimental procedures were performed according to the ethical standards of the Declaration of Helsinki and were approved by the Ethics Committee of Hiroshima University Hospital (approval no. E‐588‐2). All animal experiments were carried out in strict accordance with the recommendations of the Guide for the Care and Use of Laboratory Animals of the Hiroshima University Animal Research Committee (permission no. 29–58).

## Consent

The authors have nothing to report.

## Conflicts of Interest

The authors decalare no conflicts of interest.

## Supporting information


Data S1.

## Data Availability

This manuscript does not include any unique or meta datasets. All data are presented in the manuscript.

## References

[1] F. Bray , M. Laversanne , H. Sung , et al., “Global Cancer Statistics 2022: GLOBOCAN Estimates of Incidence and Mortality Worldwide for 36 Cancers in 185 Countries,” CA: A Cancer Journal for Clinicians 74 (2024): 229–263. 38572751 10.3322/caac.21834

[2] A. T. Lenis , P. M. Lec , K. Chamie , and M. Mshs , “Bladder Cancer,” Journal of the American Medical Association 324 (2020): 1980.33201207

[3] K. Kobatake , T. Hayashi , P. C. Black , et al., “Chronic Kidney Disease as a Risk Factor for Recurrence and Progression in Patients With Primary Non‐Muscle‐Invasive Bladder Cancer,” International Journal of Urology 24, no. 8 (2017): 594–600, 10.1111/iju.13389. 28734027

[4] A. Matsumoto , T. Nakagawa , A. Kanatani , et al., “Preoperative Chronic Kidney Disease Is Predictive of Oncological Outcome of Radical Cystectomy for Bladder Cancer,” World Journal of Urology 36 (2018): 249–256. 29185045 10.1007/s00345-017-2141-2

[5] C. Nguyen , S. Ghodoussipour , M. Winter , et al., “Chronic Kidney Disease and Radical Cystectomy for Bladder Cancer: Perioperative and Oncologic Outcomes in 1,214 Patients,” Urologic Oncology 40 (2022): 381. 10.1016/j.urolonc.2022.04.010 35599109

[6] E. Temes , S. Martin‐Puig , B. Acosta‐Iborra , et al., “Activation of HIF‐Prolyl Hydroxylases by R59949, an Inhibitor of the Diacylglycerol Kinase,” Journal of Biological Chemistry 280 (2005): 24238–24244. 15849364 10.1074/jbc.M414694200

[7] E. Temes , S. Martin‐Puig , J. Aragones , et al., “Role of Diacylglycerol Induced by Hypoxia in the Regulation of HIF‐1alpha Activity,” Biochemical and Biophysical Research Communications 315 (2004): 44–50. 15013423 10.1016/j.bbrc.2004.01.015

[8] G. Baldanzi , S. Mitola , S. Cutrupi , et al., “Activation of Diacylglycerol Kinase Alpha Is Required for VEGF‐Induced Angiogenic Signaling In Vitro,” Oncogene 23 (2004): 4828–4838. 15122338 10.1038/sj.onc.1207633

[9] R. Bacchiocchi , G. Baldanzi , D. Carbonari , et al., “Activation of Alpha‐Diacylglycerol Kinase Is Critical for the Mitogenic Properties of Anaplastic Lymphoma Kinase,” Blood 106 (2005): 2175–2182. 15928040 10.1182/blood-2005-01-0316

[10] J. Wei , J. Zhang , L. Wang , et al., “New Mouse Model of Chronic Kidney Disease Transitioned From Ischemic Acute Kidney Injury,” American Journal of Physiology. Renal Physiology 317 (2019): F286–F295. 31116604 10.1152/ajprenal.00021.2019 PMC6732455

[11] H. Moch , A. L. Cubilla , P. A. Humphrey , V. E. Reuter , and T. M. Ulbright , “The 2016 WHO Classification of Tumours of the Urinary System and Male Genital Organs—Part A: Renal, Penile, and Testicular Tumours,” European Urology 70 (2016): 93–105. 26935559 10.1016/j.eururo.2016.02.029

[12] S. B. Edge and C. C. Compton , “The American Joint Committee on Cancer: The 7th Edition of the AJCC Cancer Staging Manual and the Future of TNM,” Annals of Surgical Oncology 17 (2010): 1471–1474. 20180029 10.1245/s10434-010-0985-4

[13] K. Takemoto , K. Kobatake , K. Miura , et al., “BACH1 Promotes Clear Cell Renal Cell Carcinoma Progression by Upregulating Oxidative Stress‐Related Tumorigenicity,” Cancer Science 114, no. 2 (2022): 436–448, 10.1111/cas.15607. 36178067 PMC9899607

[14] E. R. Brooks , M. Siriruchatanon , V. Prabhu , et al., “Chronic Kidney Disease and Risk of Kidney or Urothelial Malignancy: Systematic Review and Meta‐Analysis,” Nephrology, Dialysis, Transplantation 39 (2024): 1023–1033. 10.1093/ndt/gfad249 PMC11139511 38037426

[15] F. Sakane , F. Hoshino , M. Ebina , H. Sakai , and D. Takahashi , “The Roles of Diacylglycerol Kinase Alpha in Cancer Cell Proliferation and Apoptosis,” Cancers (Basel) 13 (2021): 13205190. 10.3390/cancers13205190 PMC8534027 34680338

[16] C. L. Dominguez , D. H. Floyd , A. Xiao , et al., “Diacylglycerol Kinase α Is a Critical Signaling Node and Novel Therapeutic Target in Glioblastoma and Other Cancers,” Cancer Discovery 3 (2013): 782–797. 23558954 10.1158/2159-8290.CD-12-0215 PMC3710531

[17] K. Takeishi , A. Taketomi , K. Shirabe , et al., “Diacylglycerol Kinase Alpha Enhances Hepatocellular Carcinoma Progression by Activation of Ras‐Raf‐MEK‐ERK Pathway,” Journal of Hepatology 57 (2012): 77–83. 22425622 10.1016/j.jhep.2012.02.026

[18] K. Yanagisawa , S. Yasuda , M. Kai , et al., “Diacylglycerol Kinase Alpha Suppresses Tumor Necrosis Factor‐Alpha‐Induced Apoptosis of Human Melanoma Cells Through NF‐kappaB Activation,” Biochimica et Biophysica Acta 1771 (2007): 462–474. 17276726 10.1016/j.bbalip.2006.12.008

[19] N. Fujita , S. Hatakeyama , M. Momota , et al., “Preoperative Chronic Kidney Disease Predicts Poor Prognosis in Patients With Primary Non‐Muscle‐Invasive Bladder Cancer Who Underwent Transurethral Resection of Bladder Tumor,” Urologic Oncology 38 (2020): 684.e1. 10.1016/j.urolonc.2020.02.001 32201059

[20] I. Hamano , S. Hatakeyama , H. Iwamura , et al., “Preoperative Chronic Kidney Disease Predicts Poor Oncological Outcomes After Radical Cystectomy in Patients With Muscle‐Invasive Bladder Cancer,” Oncotarget 8 (2017): 61404–61414. 28977873 10.18632/oncotarget.18248 PMC5617433

[21] J. Li , C. Pan , A. C. Boese , et al., “DGKA Provides Platinum Resistance in Ovarian Cancer Through Activation of c‐JUN‐WEE1 Signaling,” Clinical Cancer Research 26 (2020): 3843–3855. 32341033 10.1158/1078-0432.CCR-19-3790 PMC7367757

[22] P. Russell , “Negative Regulation of Mitosis by Wee1+, a Gene Encoding a Protein Kinase Homolog,” Cell 49 (1987): 559–567. 3032459 10.1016/0092-8674(87)90458-2

[23] K. Do , J. H. Doroshow , and S. Kummar , “Wee1 Kinase as a Target for Cancer Therapy,” Cell Cycle 12 (2013): 3159–3164. 24013427 10.4161/cc.26062 PMC3865011

[24] L. Fu , R. Deng , Y. Huang , et al., “DGKA Interacts With SRC/FAK to Promote the Metastasis of Non‐Small Cell Lung Cancer,” Cancer Letters 532 (2022): 215585. 35131384 10.1016/j.canlet.2022.215585

[25] L. Fu , S. Li , W. Xiao , et al., “DGKA Mediates Resistance to PD‐1 Blockade,” Cancer Immunology Research 9 (2021): 371–385. 33608256 10.1158/2326-6066.CIR-20-0216

[26] M. Zhao , X. L. He , and X. D. Teng , “Understanding the Molecular Pathogenesis and Prognostics of Bladder Cancer: An Overview,” Chinese Journal of Cancer Research 28 (2016): 92–98. 27041931 10.3978/j.issn.1000-9604.2016.02.05 PMC4779766

[27] A. Hasan , Y. Mohammed , M. Basiony , et al., “Clinico‐Pathological Features and Immunohistochemical Comparison of p16, p53, and Ki‐67 Expression in Muscle‐Invasive and Non‐Muscle‐Invasive Conventional Urothelial Bladder Carcinoma,” Clinics and Practice 13 (2023): 806–819. 37489422 10.3390/clinpract13040073 PMC10366752

