# Supplementary material for: DGKα Enhances Tumorigenic Activity in Bladder Cancer Patients With Chronic Kidney Disease
Source: Cancer Med. 2025 Feb 27;14(5):e70710. doi: 10.1002/cam4.70710 (PMC11865707; doi:10.1002/cam4.70710)
Supplement: Supplementary file 1 — Data S1. [file CAM4-14-e70710-s001.docx]

Supplementary Table. Primer information

| Primers sequence | |  |  |
| --- | --- | --- | --- |
| Used for | Primer name |  | Sequence (5' to 3') |
| RT-qPCR | *DGKα* | Forwad | TGTGACAAAAGATGTGGTGTGT |
|  |  | Reverse | GAAGAATCGGCCTCAGCTCA |
| siRNA | si*DGKα*-1 |  | GCAUCGCAGUGCUAAACAU |
|  | si*DGKα*-2 |  | CCGGAGAAGUUCAACAGCA |
